# Prenatal bisphenol a exposure and dysregulation of infant hypothalamic-pituitary-adrenal axis function: findings from the APrON cohort study

**DOI:** 10.1186/s12940-017-0259-8

**Published:** 2017-05-19

**Authors:** Gerald F Giesbrecht, Maede Ejaredar, Jiaying Liu, Jenna Thomas, Nicole Letourneau, Tavis Campbell, Jonathan W Martin, Deborah Dewey, the APrON Study Team, B.J. Kaplan, C.J. Field, D. Dewey, R.C. Bell, F.P. Bernier, M. Cantell, L.M. Casey, M. Eliasziw, A. Farmer, L. Gagnon, G.F. Giesbrecht, L. Goonewardene, D.W. Johnston, L. Kooistra, N. Letourneau, D.P. Manca, J.W. Martin, L.J. McCargar, M. O’Beirne, V.J. Pop, N. Singhal

**Affiliations:** 1 Department of Paediatrics, University of Calgary, 2500 University Drive, Calgary, AB T2N 1N4 Canada; 2 Department of Community Health Sciences, University of Calgary, Calgary, AB Canada; 3 Department of Laboratory Medicine and Pathology, University of Alberta, Edmonton, AB Canada; 4 Department of Psychology, University of Calgary, Calgary, AB Canada; 5 Faculty of Nursing, University of Calgary, Calgary, AB Canada

**Keywords:** Bisphenol-A, Fetal exposure, Cortisol, Hypothalamic-pituitary-adrenal axis, Infant stress reactivity

## Abstract

**Background:**

Animal models show that prenatal bisphenol A (BPA) exposure leads to sexually dimorphic disruption of the neuroendocrine system in offspring, including the hypothalamic-pituitary-adrenal (HPA) neuroendocrine system, but human data are lacking. In humans, prenatal BPA exposure is associated with sex-specific behavioural problems in children, and HPA axis dysregulation may be a biological mechanism. The objective of the current study was to examine sex differences in associations between prenatal maternal urinary BPA concentration and HPA axis function in 3 month old infants.

**Methods:**

Mother-infant pairs (*n* = 132) were part of the Alberta Pregnancy Outcomes and Nutrition study, a longitudinal birth cohort recruited (2010–2012) during pregnancy. Maternal spot urine samples collected during the 2nd trimester were analyzed for total BPA and creatinine. Infant saliva samples collected prior to and after a blood draw were analyzed for cortisol. Linear growth curve models were used to characterize changes in infant cortisol as a function of prenatal BPA exposure.

**Results:**

Higher maternal BPA was associated with increases in baseline cortisol among females (β = 0.13 log μg/dL; 95% CI: 0.01, 0.26), but decreases among males (β = −0.22 log μg/dL; 95% CI: -0.39, −0.05). In contrast, higher BPA was associated with increased reactivity in males (β = .30 log μg/dL; 95% CI: 0.04, 0.56) but decreased reactivity in females (β = −0.15 log μg/dL; 95% CI: -0.35, 0.05). Models adjusting for creatinine yielded similar results.

**Conclusions:**

Prenatal BPA exposure is associated with sex-specific changes in infant HPA axis function. The biological plausibility of these findings is supported by their consistency with evidence in rodent models. Furthermore, these data support the hypotheses that sexually dimorphic changes in children’s behaviour following prenatal BPA exposure are mediated by sexually dimorphic changes in HPA axis function.

## Background

Bisphenol A (BPA) is the building block of polycarbonate plastics used to produce a wide range of consumer products including epoxy resins, thermal paper, and dental composites. Numerous rodent studies have shown that fetal/neonatal exposure to relatively low levels of BPA may result in reproductive and developmental effects, including disruption of sexual differentiation in the brain [31, 45]. These effects have led to the classification of BPA as an endocrine disruptor. Whereas the effects of perinatal BPA exposure on the development of estrogen neuroendocrine systems has been investigated extensively [37–39], the potential effect of BPA exposure on the development of the hypothalamic-pituitary-adrenal (HPA) neuroendocrine system has only recently been investigated in computer and rodent models [13, 40, 42, 43]. In this study, we provide the first human data demonstrating an association between maternal urinary total BPA concentrations during pregnancy and infant HPA axis function.

BPA is among the highest production chemicals worldwide [2], and biomonitoring studies have shown that it is commonly detected (> 82%; median concentration 0.6 – 2.6 ng/mL) in the urine of pregnant women in the USA, Canada, Mexico, and Europe [1, 9, 10, 14, 33, 47, 57]. In its report on the potential adverse reproductive and developmental effects of BPA, the National Toxicology Program at the National Institute of Environmental Health expressed concern regarding the potential effects of maternal BPA exposure on brain development and behavior in fetuses, infants and children [12]. Maternal BPA can cross the placenta into umbilical circulation [3, 32, 51] and enters breast milk [59], representing multiple pathways by which maternal BPA exposure may disrupt brain development and behavior in fetuses and infants.

Accumulating evidence suggests that prenatal BPA exposure is associated with symptoms of anxiety, depressed mood, aggression, and hyperactivity in children [18]. However, the mechanisms by which BPA exposure is associated with these behavioural outcomes are not known. The HPA axis is a potential mechanism because it mediates many effects of early life exposure on development [17], and has recently garnered some attention in relation to prenatal BPA exposure. In silico (computer) modeling has shown that BPA can bind to glucocorticoid receptors similarly to other agonists (DEXA and cortisol) [43], suggesting that BPA may be able to directly stimulate the HPA axis. Animal studies, primarily in rodents, show that prenatal BPA exposure (oral administration of 40 μg/kg of the dam’s bodyweight/day) increases basal HPA axis secretion of corticosterone in female offspring and increases HPA axis reactivity to a stressor in both males and females, with males showing significantly higher reactivity compared to females [40, 42]. Chen and colleagues [13] demonstrated that changes in HPA axis function consequent to prenatal BPA exposure mediated the effects of BPA on anxiety/depression-like behaviours in offspring. Taken together, these findings suggest that prenatal exposures to BPA may have both direct and indirect effects on the developing HPA axis and that such effects may contribute to symptoms of anxiety, depressed mood, aggression, and hyperactivity in children. Nevertheless, human data are lacking.

Recently, our research group published the first human data on the association between maternal urinary total BPA concentrations and HPA axis function [21]. This study discovered an association between BPA exposure and dysregulation of the maternal HPA axis. Specifically, higher maternal urinary total BPA concentrations during pregnancy were associated with reduced cortisol (the end product of the HPA axis in humans) at morning awakening and a flatter diurnal cortisol slope. Building upon this work, in the current study we examined the association between maternal urinary BPA concentration during pregnancy and infant HPA axis reactivity to a stressor. We hypothesized that higher maternal urinary total BPA concentrations during pregnancy are associated with changes in baseline and reactivity function of the HPA axis in infants. We furthermore hypothesized that these associations are sex-specific. To achieve these aims, maternal urinary total BPA concentration was measured during pregnancy and infant cortisol reactivity was assessed at 3 months of age.

## Methods

### Source population

Participants were 132 women enrolled in an ongoing prospective birth cohort study, the Alberta Pregnancy Outcomes and Nutrition (APrON) study (*n* = 2200), which is a community sample of volunteers recruited between 2010 and 2012. A full description of the APrON study is available elsewhere [29]. For the current analyses, women were included if they had a singleton pregnancy, were less than 27 weeks of gestation at the first study visit, were 18 years of age or older, participated in infant saliva collection at 3-months of age, and provided a second trimester urine sample. Mother-infant pairs were excluded if the mother smoked or consumed alcohol during pregnancy, or if the mother or infant were being treated with a synthetic glucocorticoid. Because of limited funding, a convenience sample of women in the full cohort was enrolled in saliva collection (*n* = 290) of which 254 completed infant saliva collection at 3-months. Of those, 132 had a 2nd trimester urine sample that could be used for the current analyses. Prior to data collection, participants provided informed consent to the procedures, which were approved by the University of Calgary Conjoint Health Research Ethics Board.

### Procedures and measures

#### Primary outcome

Infant cortisol was assessed from whole saliva samples collected before (baseline) and after (5 min, 20 min, and 40 min) an infant stressor. The stressor was a blood draw completed by a certified pediatric phlebotomist when infants were 3 months of age. Saliva was collected using sorbettes (BD Visispear™), which have been validated for cortisol collection [16] and assayed for cortisol using the Salimetrics enzyme immunoassay. It has a lower limit of sensitivity of 0.007 μg/dl, standard curve range from 0.012 to 3.0 μg/dl, and average intra- and inter-assay coefficients of variation 3.5 and 5.1%, respectively. Method accuracy, determined by serial dilution were 100.8 and 91.7%. A random 10% of samples were assayed in duplicate to confirm reliability; the intra-assay coefficient of variation and the correlation coefficient between the duplicate tests were *cv* = .035 and *r* = .99, respectively. The mean values from duplicate samples were used for analysis.

#### Primary exposure

A spot urine sample was collected into sterile urine cups from pregnant women between 14 and 26 weeks of gestation. A complete description of the methods for detecting total BPA in the current samples was reported previously [21]. Briefly, urinary total BPA concentrations were quantified by online solid-phase extraction coupled to high performance liquid chromatography and an Orbitrap Elite hybrid mass spectrometer (Thermo Fisher Scientific, San Jose, CA). The recoveries of total BPA at two levels (1 ng/mL, 10 ng/mL) were 88 and 102%, with relative standard deviations (RSD) of 11%. Linearity was evaluated over two orders of magnitude (0.5 ng/mL to 50 ng/mL, 6 point curve), and the regression coefficients of the standard curves were always >0.99. The limit of detection (LOD) was 0.32 ng/mL. For statistical analyses, total BPA concentrations below the LOD were reported as the LOD/$\sqrt{2}$.

#### Other measures

To correct for urinary dilution, urinary creatinine was quantified by laboratory services at the University of Alberta Hospital using a Synchron LX® System (Beckman Coulter). The limit of detection for creatinine was 10 mg/dL, with a dynamic range from 18 to 399 mg/dL (*r* > 0.99). Maternal sociodemographic information was collected via questionnaire during pregnancy, and infant birth outcomes were abstracted from the birth record. Prior to the blood draw, mothers were asked to verbally report on factors that could affect infant cortisol levels (i.e., sleep versus awake on the car ride to the laboratory, time since last feeding) and responses were recorded by a research assistant. Infant negative affect in the period from arrival at the laboratory until the blood draw was rated by a trained research assistant using the negative affect scale of the Behavior Rating Scales of the Bayley Scales of Infant Development 2nd edition [5], which assesses the amount and intensity of infant fussing/crying on a 5-point scale from “no negative affect displayed” to “three or more intense, heightened, or prolonged displays of negative affect”.

#### Statistical analysis

Descriptive statistics are reported as geometric means and medians for continuous variables and percentages for categorical variables. Differences in demographic and study variables across maternal BPA tertile (Table 1) and infant sex (Table 2) were calculated by ANOVA or chi-squared, as appropriate. The hypotheses were tested using the MIXED procedure in SPSS version 22.0. Linear growth curve models were estimated to model the change in infant cortisol from baseline through 40 min post stressor (i.e., cortisol reactivity) as a function of maternal total BPA concentration during pregnancy. The outcome of these models was infant cortisol concentration, which was natural log transformed. At level 1, both the linear and quadratic effects of time were included to allow curvilinear trajectories in infant cortisol (cortisol increases are expected to slow or reverse between 20 and 40 min posts stressor) and to allow for individual differences in peak cortisol (maximum levels are delayed in some infants). Time was centered at baseline, thus the intercept in the growth curve models refers to infant cortisol concentration at baseline. Cortisol reactivity was determined by the slope parameter estimates for the linear and quadratic effects of time. The primary variables of interest were maternal total BPA concentration and the interaction term between maternal BPA and fetal sex; these were included in the level 2 submodel for the intercept and linear slope. Maternal total BPA concentration was centered at the sample mean and sex was centered at females. Because cortisol was natural log transformed, interpretation of the beta coefficients is facilitated by expressing each coefficient as a percent change using the formula β% change = (e^β^ − 1) × 100. The percent change can be thought of as an effect size because it refers to the size of the change in infant cortisol for each 1 unit change in the predictor (holding all other variables constant).

**Table 1:** Participant characteristics and descriptive statistics by BPA tertile

	Lower tertile (*n* = 44)			Middle tertile (*n* = 44)			Upper tertile (*n* = 44)		
Maternal Variable	GM(SE)	Median	Range	GM(SE)	Median	Range	GM(SE)	Median	Range
Age (years)	29.7 (.61)	30	24–40	30.8 (.56)	31.5	20–39	31.3 (.63)	32	22–42
Pre-pregnancy BMI	24.6 (.65)	24	19.8–42.3	25.4 (.82)	25	18.4–38.5	25.9 (.93)	24	19.1–43.1
Total BPA (ng/mL)	.34 (.02)	.34	.16–.60	.92 (.04)	.93	.63–1.40	3.84 (1.58)	3.15	1.43–43.42
Creatinine (mmol/L)	4.01 (.44)	4.10	.80–12.40	8.06 (.72)	9.10	2.30–19.10	11.96 (.94)	13.10	3.90–32.80
Infant Variable									
Gestational age at birth (weeks)	39.5 (.19)	40.0	35.3–41.6	39.0 (.27)	39.2	33.0–41.3	39.2 (.21)	39.0	35.6–41.7
Birthweight (grams)	3501 (74)	3418	2725–4904	3254 (86)	3410	2030–4420	3329 (75)	3385	2220–4200
Baseline cortisol (μg/dL)	.25 (.03)	.26	.05–1.02	.25 (.04)	.26	.06–1.51	.22 (.04)	.22	.10–1.17
	Percent			Percent			Percent		
Household Income									
< $20,000	0			4.5			0		
$20 k – $40 k	2.4			2.3			9.1		
$40 k – $70 k	11.9			11.4			11.4		
$70 k – $100 k	28.6			22.7			25.0		
> $100,000	57.1			59.1			54.5		
Education									
High school diploma	14.3			6.8			4.5		
College diploma	11.9			15.9			34.1		
University degree	73.8			77.3			61.4		
Ethnicity									
White	86.4			77.3			86.4		
Other	13.6			22.7			13.6		
Marital status									
Married	90.7			81.8			90.9		
Common law	9.3			15.9			9.1		
Single/separated/ divorced	0			2.3			0		
Nulliparous	52.3			47.7			61.4		
Infant Sex									
Male	50			50			50		

Note. *GM* geometric mean, *SE* standard error of the mean

**Table 2:** Participant characteristics and descriptive statistics by child sex

	Males (*n* = 66)			Females (*n* = 66)		
Maternal Variable	GM(SE)	Median	Range	GM(SE)	Median	Range
Age (years)	31.1 (.47)	31	24–40	30.1 (.50)	31	20–42
Pre-pregnancy BMI	24.8 (.65)	24	18.4–42.2	25.8 (.67)	25	19.1–43.1
Total BPA (ng/mL)	1.10 (.97)	.89	.16–43.42	1.04 (.63)	.98	.23–39.84
Creatinine (mmol/L)	6.74 (.73)	7.65	.80–32.80	7.87 (.74)	8.20	.90–24.70
Infant Variable						
Gestational age at birth (weeks)	39.2 (.19)	39.3	33.0–41.7	39.3 (.17)	39.9	35.3–41.4
Birthweight (grams)	3448 (72)	3510	2030–4904	3273 (54)	3352	2215–4200
Baseline cortisol (μg/dL)	.25 (.02)	.24	.05–1.03	.24 (.03)	.23	.06–1.51
	Percent			Percent		
Household Income						
< $20,000	1.6			1.5		
$20 k – $40 k	6.3			3.0		
$40 k – $70 k	4.7			18.2		
$70 k – $100 k	23.4			27.3		
> $100,000	64.1			50.0		
Education						
High school diploma	6.3			10.6		
College diploma	14.1			27.3		
University degree	79.8			62.1		
Ethnicity						
White	80.3			86.4		
Other	19.7			13.6		
Marital status						
Married	92.3			83.3		
Common law	7.7			15.2		
Single/separated/divorced	0			1.5		
Nulliparous	53.0			54.5		

Note. *GM* geometric mean. *SE* standard error of the mean

#### Covariates

Maternal urinary total BPA was adjusted for creatinine in statistical models in two ways to reflect current practice [4]: one was to add creatinine as an independent variable to the model (creatinine as covariate) and the other was to divide BPA by creatinine concentration (creatinine-corrected). Additional potential covariates that had been identified in previous studies [11, 23, 24] were considered as potential confounders of the maternal BPA – infant cortisol association. Selection of covariates, random effects and error structure was based upon model fit as assessed by −2 log likelihood and Bayesian Information Criterion (BIC), except for infant gestational age at birth which was included because it is associated with infant HPA axis function. In preliminary models, infant age at assessment, infant birthweight, infant sleeping versus awake on the car ride to the appointment, infant behavioral fussiness at baseline, time of day at baseline, maternal or infant medication use, and maternal pre-pregnancy BMI did not improve model fit and were therefore excluded from further analysis. Covariates that were retained included gestational week of pregnancy for maternal urinary BPA collection, time of day at which maternal urine was collected, and infant gestational age at birth.

#### Sensitivity analyses

Because gestational age at birth and birthweight are associated with infant HPA axis function [11, 23] and prenatal BPA exposure [6, 52], we conducted sensitivity analyses excluding infants with low birthweight (< 2500 g) or preterm birth (< 37 weeks gestation). To determine the extent to which urinary dilution may affect the association between BPA and cortisol, all analyses were re-run excluding women who had urinary creatinine concentrations that were considered too diluted (< 2.65 mmol creatinine/L) or too concentrated (> 26.5 mmol creatinine/L) according to the World Health Organization guidelines for occupational monitoring, which have also been applied to non-occupational studies [4].

## Results

### Descriptive findings

Overall, the participants were relatively well-educated (71% had a university education), married or living in common-law relationships (99%), mature (mean age = 30.6 years, range 20-42 years) and nulliparous (54%) at enrollment. The majority of women (82%) lived in households with annual income greater than $70,000 CAD (according to Statistics Canada, the median household income within the recruitment region was $98,030), and most self-identified as White (83%). Infants averaged 12.5 weeks old at time of assessment (range 9.1 – 16 weeks). The sex ratio of infants was equal (66 m: 66f).

As the sample was embedded within a larger cohort study, we tested for potential selection bias by comparing the characteristics of women included in this study with the full APrON cohort. Women in the current analysis had higher pre-pregnancy BMI compared to the full cohort (25.7 kg/m^2^ versus 24.1 kg/m^2^; *p* < .05), but did not differ from the full cohort on income, ethnicity, age, parity, or marital status (all *p* values > .05).

Characteristics of the participants by BPA tertile are listed in Table 1 and by sex in Table 2. Table 1 reveals that creatinine concentrations increased by BPA tertile, *F*(2, 129) = 34.5, *p* < .001. All other variables did not differ between groups (*p* > 0.05). Table 2 shows that, as expected, female infants had lower birthweight compared to male infants, *F*(2130) = 4.7, *p* = .03. All other variables did not differ between groups.

Total BPA was detected above the LOD in 90.9% of the maternal urine samples. Average BPA concentrations (GM = 1.07 ng/mL, range .16 – 43.4 ng/mL) were within the range previously reported for pregnant women in North America. Creatinine was detected in all samples. Cortisol was detected in all samples, however, two samples yielded results that were not biologically plausible (> 3 μg/dL) [25, 35, 50], and were removed.

### Associations between maternal total BPA and infant baseline cortisol

The association between maternal total BPA concentration and infant cortisol was not significant for baseline, β = 0.002 (95% CI: -0.08, 0.08), when infant sex was not considered in the model (see Model 1, Table 3). However, when sex of the infant, and the interaction term between sex and total BPA concentration, were added to the model, significant conditional associations were observed (see Model 2, Table 3). Specifically, higher maternal total BPA was associated with a significant increase in baseline salivary cortisol among female infants, β = 0.13 (95% CI: 0.01, 0.26), corresponding to a 14% increase per one unit increase in maternal total BPA (note that total BPA was log10 transformed therefore 1 unit = a 10-fold increase). The association between maternal total BPA and baseline infant cortisol was significantly different for male and female infants, β = −0.22 (95% CI: -0.39, −0.05). In contrast to the *increase* in baseline cortisol observed in female infants, each 10-fold increase in maternal total BPA was associated with an 8.1% *decrease* in baseline cortisol for male infants.

**Table 3:** Model estimates for the association between maternal total urinary BPA (log10 ng/mL) concentration and infant salivary cortisol concentration (natural log μg/dL): *n* = 132

	Model 1: BPA		Model 2: BPA with sex		Model 3: BPA with sex and creatinine	
Fixed Effects	β Coefficient (95% CI)	% change (95% CI)	β Coefficient (95% CI)	% change (95% CI)	β Coefficient (95% CI)	% change (95% CI)
INTERCEPT (baseline cortisol)	.31 (.12, .49)	GM = .37 μg/dL at baseline	.31 (.12, .50)	GM = .38 μg/dL at baseline for females	.31 (.12, .50)	GM = .38 μg/dL at baseline for females
Time of day for urine collection (hours)	.001 (−.006, .008)		.002 (−.005, .009)		.002 (−.005, .009)	
Gestational age at urine collection (weeks)	−.004 (−.01, .003)		−.005 (−.01, .003)		−.005 (−.01, .003)	
Gestational age at birth (weeks)	−.005 (−.02, .01)		−.006 (−.02, .01)		−.005 (−.02, .01)	
Creatinine (mmol/L)					.0001 (−.005, .005)	
Infant Sex			−.02 (−.07, .03)		−.02 (−.07, .03)	
BPA (log10 ng/mL)	.002 (−.08, .08)	.2% (−.8, .8) increase per 10-fold increase in BPA at baseline	.13 (.01, .26)	14% (.7, 30) increase per 10-fold increase in BPA at baseline for females	.13 (−.01, .28)	14% (−1, 32) increase per 10-fold increase in BPA at baseline for females
BPA X sex			−.22 (−.39, −.05)	−20% (−32, −5) difference between males and females; net effect is 8.1% decrease per 10-fold increase in BPA at baseline for males	−.22 (−.39, −.05)	−20% (−32, −5) difference between males and females; net effect is a 9% decrease per 10-fold increase in BPA at baseline for males
TIME ^2^ (hours)	−.33 (−.47, −.18)		−.33 (−.48, −.18)		−.33 (−.48, −.18)	
TIME (hours)	.31 (.19, .44)	37% (21, 55) increase per hour	.32 (.19, .46)	38% (21, 58) increase per hour for females	.32 (.19, .45)	38% (21, 57) per hour for females
Gestational age at birth (weeks)	−.02 (−.04, .01)		−.02 (−.04, .01)		−.02 (−.04, .01)	
Creatinine (mmol/L)					.003 (−.005, .01)	
Infant Sex			−.03 (−.11, .05)	−3% (−10, 5) difference between males and females; net effect is 34% increase per hour in males	−.02 (−.11, .06)	−3% (−10, 5) difference between males and females; net effect is 34% increase per hour in males
BPA (log10 ng/mL)	.02 (−.11, .15)	2% (−10, 17) per hour per 10-fold increase in BPA	−.15 (−.35, .05)	−14% (−30, 5) decrease per hour for females per 10-fold increase in BPA	−.20 (−.43, .03)	−18% (−35, 3) decrease per hour for females per 10-fold increase in BPA
BPA X sex			.30 (.04, .56)	35% (4, 75) difference between males and females; net effect is a 17% per hour increase in males per 10-fold increase in BPA	.33 (.06, .60)	39% (6, 82) difference between males and females; net effect is a 15% per hour increase in males per 10-fold increase in BPA

Note. Time was centered at baseline; sex was centered at female; all other variables were centered at the grand mean. Because total BPA was log10 transformed, the interpretation of the estimates are per 10-fold change in BPA

### Associations between maternal total BPA and infant cortisol reactivity

**Fig. 1:**
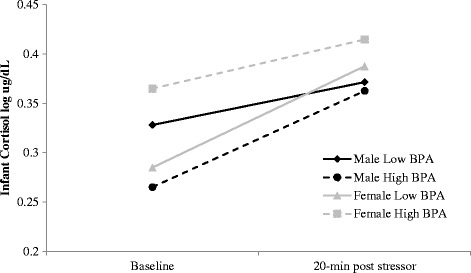
Infant baseline cortisol and cortisol reactivity as a function of maternal urinary total BPA concentration and infant sex

There was no association between maternal total BPA concentration and infant cortisol reactivity (i.e., Time), β = 0.02 (95% CI: -0.11, 0.15) when infant sex was not considered in the model (see Model 1, Table 3). With sex added to the model, the association between maternal total BPA and infant cortisol reactivity was non-significant for female infants, β = −0.15 (95% CI: -0.35, 0.05), however the association differed for male and females infants, β = .30, (95% CI: 0.04, 0.56), indicating that the association between maternal BPA and infant cortisol reactivity was dependant on infant sex. For female infants, each 10-fold increase in maternal BPA was associated with a 14% *reduction* in cortisol reactivity, whereas the same increase was associated with a 17% *increase* in cortisol reactivity among male infants. To illustrate these associations, the estimates obtained in Model 2 have been plotted (Fig. 1) at the mean of the upper and lower quartiles for BPA to represent the highest and lowest exposures in the sample.

Results after adjusting for creatinine (see Model 3, Table 3) were similar to those reported in Model 2. However, the association between maternal BPA and cortisol at baseline for females was reduced to a non-significant effect, β = 0.13 (95% CI: -0.01, 0.28), and the associations between BPA and reactivity and sex differences in the associations between BPA and reactivity become slightly larger. One additional model (results not shown) with BPA corrected for creatinine revealed comparable, although slightly smaller, effect estimates relative to those observed in Models 2 and 3, and the standard errors in this model were slightly larger. All confidence intervals for effect estimates spanned zero in this model.

### Sensitivity analysis

To determine whether infants who were born preterm (< 37 weeks gestation) or low birthweight (< 2500 g) may be contributing to our findings, the analyses were redone with nine infants excluded. The findings reported in Table 3 remain unchanged after these exclusions (data not shown). To determine whether urinary dilution/concentration may have affected the results, we excluded women with low (*n* = 14) or high (*n* = 1) creatinine concentrations (using the WHO criteria defined above). Again, the findings reported in Table 3 remain unchanged after these exclusions (data not shown).

## Discussion

To our knowledge, these data are the first to demonstrate an association between maternal urinary total BPA concentrations during pregnancy and offspring HPA axis function in a human population. The data indicated that associations between prenatal maternal BPA and infant cortisol were dependent on sex. For females, elevated maternal BPA was associated with elevated baseline cortisol, whereas in males it was associated with decreased baseline cortisol. Furthermore, greater maternal urinary total BPA concentration was associated with attenuation of infant cortisol reactivity in female infants but potentiation of cortisol reactivity in male infants. These findings suggest that fetal/infant sex plays a critical role in the association between maternal BPA concentration and children’s HPA axis function.

The sex differences observed in these data recapitulate findings in rodent models. Female, but not male, rodents had increased basal corticosterone levels following perinatal BPA exposure but males had exaggerated corticosterone reactivity to a stressor [40, 42]. Although exaggerated corticosterone reactivity was also observed in females, the effect in males was significantly larger than the effect in females. The consistency of the human data with animal evidence supports the biological plausibility of these findings. Note however that not all animal studies have been consistent – Chen and colleagues observed elevations in basal and reactivity corticosterone measure only in males. Differences between studies could be related to factors such as dose and age of testing.

It is important to note that the sex differences we observed cannot be accounted for by pre-existing sex differences in baseline cortisol or cortisol reactivity. When we removed BPA from the statistical model, there were no sex difference in baseline cortisol, β = −.01 (95% CI: -0.06, 0.04), or in cortisol reactivity, β = −.03 (95% CI: -0.11, 0.05). Sex differences in baseline cortisol and cortisol reactivity became apparent only as a function of prenatal BPA exposure.

Recently, Chen and colleagues [13] reported that BPA-related changes in the offspring HPA axis in rats were responsible for increases in anxiety/depression-like behaviours, possibly through epigenetic changes in the hypothalamus and hippocampus [30]. It is well accepted that alteration of HPA axis function as a consequence of prenatal and early life exposure is a primary mechanism by which such exposures have long-lasting effects on behavior and development. Our findings are consistent with the hypothesis that BPA-related behavioral changes observed in human children may be accompanied by changes in the infant HPA axis. This hypothesis is supported both by the well-established role of the HPA axis in developmental psychopathology [34] and the frequently observed sex differences in behavioral outcomes of children prenatally exposed to BPA [9, 20, 27, 41, 44].

Although previous studies have not examined HPA axis function in human offspring as a function of prenatal BPA exposure, several studies have examined behavioral outcomes. These studies have consistently reported sex differences, however the direction of these sex differences was not consistent across studies. Some studies suggest that perinatal BPA exposure is associated with increased internalizing and externalizing behaviors in females [8, 9], whereas others report increases in these behaviors among males [20, 27, 41, 44]. Such inconsistencies across studies may be related to the timing of exposure, as suggested by the findings of Roen and colleagues, who reported contrasting findings for prenatal versus postnatal BPA exposure. Prenatal exposure was associated with increased behavioural problems in boys but not girls whereas postnatal exposure was associated with increased behavioural problems in girls but not boys. Further research is needed to clarify the pattern of sex differences in the associations between BPA exposure and behavioral outcomes in children, and to determine what role the HPA axis function may play in these outcomes.

Although the mechanisms by which prenatal BPA exposure alters HPA axis function are not conclusively known, several potential routes have been identified. First, BPA, like other agonists (i.e., dexamethasone and cortisol) can bind to glucocorticoid receptors [43], making plausible direct stimulation of the HPA axis through BPA binding to glucocorticoid receptors. Second, BPA may disrupt the effects of sex hormones on the development and function of the HPA axis. Sex differences in HPA axis function appear to be modulated by the gonadal steroid environment, acting in part by modulating adrenal, anterior pituitary, and hypothalamic functions [26]. Given that BPA is an estrogenic endocrine disruptor [58] and that estrogen plays a critical role in HPA axis regulation [55], it is plausible that changes in estrogen receptors following BPA exposure influence HPA axis development and function. The involvement of gonadal steroids may also help to explain sex differences in the effects of BPA on behavioural outcomes. Third, BPA may alter placental metabolism of endogenous maternal cortisol by increasing the expression of 11β-hydroxysteroid dehydrogenasetype 1 (11β-HSD1) [54], an enzyme that preferentially converts the inactive hormone cortisone to the active hormone cortisol [7, 22, 56]. This change of placental metabolism would potentially increase fetal exposure to maternal cortisol, and thereby alter infant HPA axis function and potentially, child behaviour. Further research that assesses prenatal BPA exposure, infant HPA axis function, and child behaviour is needed to elaborate the potential pathways by which BPA exposure can influence child behaviour.

It is important to note that the exposures observed in the current sample are well below the exposures that animals receive in laboratory studies, even in so-called low dose studies, and below the Tolerable Daily Intakes (TDIs) established by Canadian health authorities. BPA exposure in the Canadian population is likely well below the provisional TDIs established by the Food Directorate of Health Canada (25 μg/kg bw/day) [19, 28]. Based on data from Covaci and colleagues [15], where daily intake was calculated from BPA concentrations in spot urine samples of mothers, we estimate that the highest daily intake in the current sample ranges from 0.9 - 1.1 μg/kg bw/day. The fact that associations with infant HPA axis function can be detected even at exposure levels well below the most stringent (European) TDIs suggests that such exposures may play a role in shaping the development of the infant HPA axis.

The clinical significance of these findings is not clear given the lack of normative data on cortisol reactivity in young infants. Nevertheless, it may be possible to evaluate the clinical significance of these findings by comparison to data on dampening of the cortisol response using sucrose following inoculations [36] or lidocaine prior to circumcision [48], both of which result in an approximately 30% reduction in cortisol relative to baseline or control, respectively. Note however that there are few studies and not all have observed significant effects, and while sucrose is now recommended for infant inoculations [46], the most convincing evidence comes from behavioural rather than physiologic data. The current study observed an approximately 37% increase in cortisol per hour as a function of each 10-fold increase in prenatal BPA exposure, and there was an 11.3-fold higher BPA concentration in the upper compared to the lower tertile (see Table 1). Given that cortisol tends to peak at about 30 min post-stressor, these data suggest that infants in the upper tertile of BPA exposure had 20% larger increases in cortisol over the first 30 min following the blood draw compared to infants in the lower tertile of exposure. Further study is required to determine whether BPA-related increases in cortisol are associated with clinically relevant behavioral or health outcomes.

### Strengths and limitations

This study had several strengths and limitations. To our knowledge, it is the first human study to examine the association between maternal urinary total BPA and HPA axis function in children. With respect to limitations, the sample size was modest and it will be important to replicate these findings in a larger study to confirm these associations. Although sample size was not a concern for those effects that were significant, it will be important to replicate these findings in a larger sample to confirm these associations. Second, we used a single measure of maternal urinary BPA in mid-pregnancy as our exposure variable. BPA has relatively short half-life following oral exposure [49, 53] and there is inherent variability in BPA concentrations over time, which pregnancy may accentuate [9]. Accordingly, a single spot urine sample may not provide a complete estimate of fetal in utero exposure to BPA. Nevertheless, to reduce measurement variability associated with a single sample, we included in our statistical models the gestational week and the time day at which the urine sample was collected, as suggested by Braun and colleagues [9]. Further, any potential measurement bias resulting from a single urine sample would likely be non-differential, as the measurement error applies equally to both lower and higher BPA concentrations, and any measurement bias would likely decrease the association between BPA urinary concentrations and infant cortisol. Thus, the effects observed here are more likely to underestimate rather than overestimate the true association between maternal BPA and offspring cortisol. Third, although we tested many potential covariates and included those that were theoretically important and statistically related to the outcome in our models, the existence of residual confounding in our findings cannot be disregarded. Finally, considering that the APrON study population is largely White, more highly educated, and has higher income compared to the Canadian population [29], generalization to other ethnic and socioeconomic groups should be made with caution. Nevertheless, we expect on the basis of our sample that these findings apply to the majority of middle class women and infants living in the developed world.

## Conclusion

Our results suggest that prenatal exposure to BPA is associated with sex-specific changes in infant HPA axis function. Given that HPA axis dysfunction is a presumed mechanism underlying developmental psychopathology and behavioral problems in children, we propose that sexually dimorphic associations between prenatal BPA exposure and behavioral outcomes in children are mediated, at least in part, by sex-specific dysregulation of the HPA axis.

## Abbreviations

APrON: Alberta Pregnancy Outcomes and Nutrition
BIC: Bayesian Information Criterion
BPA: bisphenol A
HPA: hypothalamic-pituitary-adrenal
